# Rapid and sensitive detection of *Mycobacterium tuberculosis* using the RPA/Cas12f1_ge4.1 system with fluorescence and lateral flow readouts

**DOI:** 10.1128/spectrum.02652-24

**Published:** 2025-06-09

**Authors:** Zhongliang Deng, Xingyong Weng, Honghua Tang, Tintao Zou, Xuan Zhou, Hangxi Liu, Piaoting Wen, Gemiao Luo, Tian Gan, Jun He

**Affiliations:** 1 The Affiliated Nanhua Hospital, Department of Clinical Laboratory, Hengyang Medical School, University of South China 34706, Hengyang, Hunan, China; 2 Department of Public Health Laboratory Sciences, College of Public Health, Hengyang Medical School, University of South China, Hengyang, Hunan, China; 3 Laboratory Department of Yiyang Disease Prevention and Control Center, Yiyang, Hunan, China; 4 The First Affiliated Hospital of University of South China, Hengyang Medical School, University of South China Hengyang Medical School 159373, Hengyang, Hunan, China; FIND, Geneva, Switzerland

**Keywords:** UnCas12f1, recombinase polymerase amplification (RPA), *Mycobacterium tuberculosis*, RPA/Cas12f1_ge4.1, fluorescence, lateral flow readouts

## Abstract

Tuberculosis remains a major global health threat, with existing detection methods often limited by efficiency and resource demands. Our previous PAM-dependent dsDNA Target-activated Cas12f1 *Trans* Reporter (PDTCTR) fluorescence sensing platform, while effective for PAM-dependent pathogen detection, was constrained by its reliance on specialized fluorescence equipment and lack of visual output, limiting its use in resource-limited settings. To overcome these limitations, we introduce an innovative RPA/CRISPR-Cas12f1_ge4.1 dual-mode system for rapid *Mycobacterium tuberculosis* detection. This system combines engineered Cas12f_ge4.1 with recombinase polymerase amplification (RPA), offering both fluorescent and lateral flow detection. It achieves high sensitivity with detection limits of 10 copies/µL (fluorescence) and 100 copies/µL (lateral flow), alongside 100% specificity. In clinical validation, compared with a commercial qPCR kit, the fluorescent and lateral flow approaches demonstrate sensitivities of 94.52% (69/73, 95% confidence interval [CI]: 85.84%–98.23%) and 90.41% (66/73, 95% CI: 80.67%–95.73%), respectively, while maintaining 100% (40/40, 95% CI: 89.09%–100%) specificity and high concordance (kappa values: 0.924 and 0.878). Detection is completed within 1 h, providing a rapid, sensitive, and specific solution for *M. tuberculosis* identification. This dual-mode capability represents a significant advancement in current tuberculosis diagnostics, enabling both sensitive laboratory confirmation and rapid point-of-care screening. Our versatile and efficient method promises to transform tuberculosis diagnostics, particularly in resource-constrained environments.

**IMPORTANCE:**

Tuberculosis (TB) remains a significant global health challenge, demanding rapid and accurate detection for effective management. The innovative RPA/CRISPR-Cas12f1_ge4.1 dual-mode system represents a major advancement in TB diagnostics, offering highly sensitive and specific detection of *Mycobacterium tuberculosis* DNA. This adaptable system, incorporating both fluorescent and lateral flow detection modes, is designed for use in both advanced laboratories and resource-limited settings. Its high performance, rigorously validated through clinical trials, holds the potential to revolutionize TB diagnosis, particularly in high-burden, low-resource areas. By facilitating earlier treatment and enhancing control of TB transmission, this system could significantly contribute to global efforts in combating this persistent public health threat.

## INTRODUCTION

Tuberculosis (TB), caused by *Mycobacterium tuberculosis*, remains a significant global health challenge, with an estimated 10.8 million new infections reported in 2023 alone (1, 2). Despite advancements in diagnostics technologies, approximately 30% of TB cases go undetected, underscoring the urgent need for improved detection methods (3). Current diagnostic approaches, including sputum smear microscopy, culture techniques, and the Xpert MTB/RIF assay, are limited by factors, such as complexity, extended turnaround times, and suboptimal sensitivity and specificity (4–6). Consequently, there is a pressing demand for early, rapid, sensitive, and accurate *M. tuberculosis* detection methods to facilitate timely treatment and prevent disease transmission.

The advent of the CRISPR/Cas system has revolutionized molecular biology and diagnostics. In particular, CRISPR/Cas12 and Cas13 systems have emerged as promising candidates for next-generation molecular diagnostics due to their target-activated collateral cleavage activities (7, 8). While several CRISPR-Cas-based platforms for *M. tuberculosis* detection have been developed, including Cas12a-based MCMD (9), Cas12b-based TB-QUICK (10), and Cas13a-based systems (11), they continue to face challenges in single nucleotide polymorphism (SNP) discrimination, nuclease thermostability, and *in vitro* transcription operations.

The discovery of Cas14a1 (Un1Cas12f1) by Harrington et al. in 2018 marked a significant advancement in CRISPR technology (12). This miniaturized nuclease demonstrates exceptional single-base resolution and specificity in SNP detection, surpassing the capabilities of Cas12 (13). Although wild-type Cas12f1 showed initial promise, its efficiency in mammalian systems was initially low (14, 15). Kim et al. addressed this limitation by engineering the Cas12f1_ge4.1 system, significantly enhancing its *in vivo* gene editing and transcriptional control capabilities (16). However, existing CRISPR/Cas14-based diagnostic platforms, such as Cas14-DETECTR and ATCas RNA (17, 18), still face challenges in clinical applications due to their reliance on complex single-strand preparation or *in vitro* transcription processes. Notably, the potential of the engineered Cas12f1_ge4.1 system for molecular diagnostics remained largely unexplored.

Building upon these advancements, we developed the PDTCTR platform, which integrates the optimized CRISPR/Cas12f1_ge4.1 system with RPA (19). This system enables rapid and highly sensitive pathogen detection, with sensitivity improved 100-fold compared with the wild-type system, while demonstrating excellent single-base discrimination capability. Although PDTCTR offers operational simplicity and direct double-stranded DNA detection advantages, the fluorescence-based sensing platform requires central laboratories, sophisticated instrumentation, skilled operators, and specific environmental conditions, resulting in higher testing costs and limiting widespread adoption in resource-limited regions.

To address these limitations, we have developed an innovative dual-modal detection system integrating RPA with CRISPR-Cas12f1_ge4.1 for the rapid and sensitive early detection of *M. tuberculosis* DNA. This advanced platform synergistically combines the exquisite target specificity of Cas12f1_ge4.1, with the efficient isothermal amplification capabilities of RPA, facilitating detection through either portable fluorescence readers or simple visual assessment via colloidal gold lateral flow strips. By incorporating the inherent advantages of lateral flow technology—including cost-effectiveness, operational simplicity, and rapid result generation—our system demonstrates enhanced versatility, making it suitable for implementation in both sophisticated laboratory environments and resource-constrained settings where diagnostic infrastructure is limited.

In this study, we present the development, optimization, and validation of this novel dual-mode system for *M. tuberculosis* detection. We demonstrate its superior sensitivity, specificity, and adaptability across various healthcare settings. Our work aims to improve TB management and reduce global health burdens, particularly in resource-constrained environments, potentially revolutionizing the approach to TB diagnosis and control worldwide.

## MATERIALS AND METHODS

### Reagents and materials

*M. tuberculosis* reference strain H37Rv and clinical isolates were obtained from the Department of Laboratory Medicine, Affiliated Nanhua Hospital, Nanhua University. Additional bacterial strains used for specificity testing included *M. kansasii*, *M. chelonae*, *M. avium*, *M. fortuitum*, *M. gordonae*, *M. asiaticum*, *M. pneumoniae*, *S. pneumoniae*, *H. influenzae*, and *K. pneumoniae*.

The QIAamp Blood Mini Kit was purchased from QIAGEN (Hilden, Germany). RPA reagents were obtained from TwistDx Ltd. (Cambridge, UK). The MEGAscript Kit was from Thermo Scientific (AM1334). HybriDetect Dipsticks were acquired from Milenia Biotec GmbH (Germany). All other chemicals were of analytical grade and used without further purification.

### DNA extraction and preparation

Genomic DNA was extracted from bacterial cultures and sputum samples using the QIAamp Blood Mini Kit (QIAGEN, Hilden, Germany) following the manufacturer’s protocol. Clinical sputum samples were pre-treated with 4% NaOH (2–3 volumes) for decontamination and liquefaction prior to extraction. DNA concentration and purity were assessed using a NanoDrop spectrophotometer.

### RPA primer and sgRNA design

RPA primers targeting *M. tuberculosis* insertion sequences IS6110 (GenBank: X17348.1) and IS1081 (GenBank: CP003248.2:1341284–1342995) were designed using Primer Premier five software and validated for specificity via BLAST analysis (Table S1). Five sgRNAs for each target gene were designed as detailed in Table S2.

### Recombinase polymerase amplification (RPA)

RPA reactions (50 µL) contained 2.4 µL each of forward and reverse primers (10 µM), 29.5 µL rehydration buffer, lyophilized RPA enzymes (TwistDx Ltd., Cambridge, UK), 2 µL template DNA, and 2.5 µL magnesium acetate (280 mM). Reactions were incubated at 42°C for 20 min.

### sgRNA preparation

sgRNAs were prepared by PCR amplification of a pUC57-sgRNA plasmid template containing a T7 promoter, followed by *in vitro* transcription using the MEGAscript Kit (Thermo Scientific, AM1334). Transcription products were verified by 2% agarose gel electrophoresis.

### RPA/CRISPR-Cas12f1_ge4.1 fluorescence assay

Cas12f1 ribonucleoprotein (RNP) complexes were formed by incubating 150 nM sgRNA with 100 nM Cas12f1 in assembly buffer (10 mM Tris–HCl [pH 7.5], 100 mM NaCl, 1 mM EDTA, 1 mM DTT) at 37°C for 30 min. The RNP complex was combined with RPA products, 200 nM fluorescence quenching probe (5′-FAM-TTTTTTTTTTTT-BHQ1-3′), and reaction buffer (10 mM Tris–HCl [pH 7.5], 100 mM NaCl, 1 mM DTT, 10 mM MgCl_2_). Fluorescence was monitored in real-time at 46°C using a fluorescence spectrometer.

### RPA/CRISPR-Cas12f1_ge4.1 lateral flow assay

For lateral flow detection, a biotin-labeled reporter molecule (5′-FAM-TTTTTTTTTTTT-Biotin-3′) was used. The CRISPR-Cas12f_ge4.1 trans-cleavage reaction product (10 µL) was applied to HybriDetect Dipsticks (Milenia Biotec GmbH, Germany) and immersed in 100 µL of HybriDetect Assay Buffer. Results were visually interpreted within 2 min.

### Sensitivity and specificity evaluation

To determine the sensitivity of the RPA/Cas12f1_ge4.1 system, *M. tuberculosis* H37Rv genomic DNA was serially diluted to concentrations of 500 pg/µL, 50 pg/µL, 5 pg/µL, 500 fg/µL, 50 fg/µL, and 5 fg/µL. These dilutions corresponded to approximately 10^5^, 10^4^, 10^3^, 10^2^, 10, and 1 copy/µL, respectively. DNA copy numbers were calculated using the formula: copies/µL = (6.02 × 10²³) × (DNA concentration [ng/µL] ×10⁻⁹) / (genome size [bp] × 660 [g/mol/bp]) assuming a genome size of 4.4 Mbp for *M. tuberculosis* H37Rv. For each assay, 2 µL of each dilution was used as template. All experiments were performed in triplicate to evaluate the reproducibility of the RPA/Cas12f1_ge4.1 detection system.

Specificity was assessed against a panel of nontuberculous mycobacteria (NTM) and common respiratory pathogens, including *M. kansasii*, *M. chelonae*, *M. fortuitum*, *M. gordonae*, *M. avium*, *M. asiaticum*, *M. pneumoniae*, *S. pneumoniae*, *H. influenzae*, and *K. pneumoniae*. Both fluorescence and lateral flow assays were performed for each organism.

### Clinical validation

We analyzed 113 clinical sputum samples (73 *M. tuberculosis*-positive and 40 *M*. *tuberculosis*-negative) using the RPA/Cas12f1_ge4.1 system. Results were compared with qPCR outcomes. The study was approved by the Ethics Committee of the Affiliated Nanhua Hospital (approval number: 2024-ky-051), and all participants provided informed consent.

### Statistical analysis

Data were analyzed using SPSS 25.0 software. Results are expressed as mean ± standard deviation (SD). Independent samples *t*-tests were used for group comparisons, with *P* < 0.05 considered statistically significant. Kappa tests assessed consistency between detection methods.

## RESULTS

### Development of the RPA/CRISPR-Cas12f1_ge4.1 dual-mode detection system

**Fig 1:**
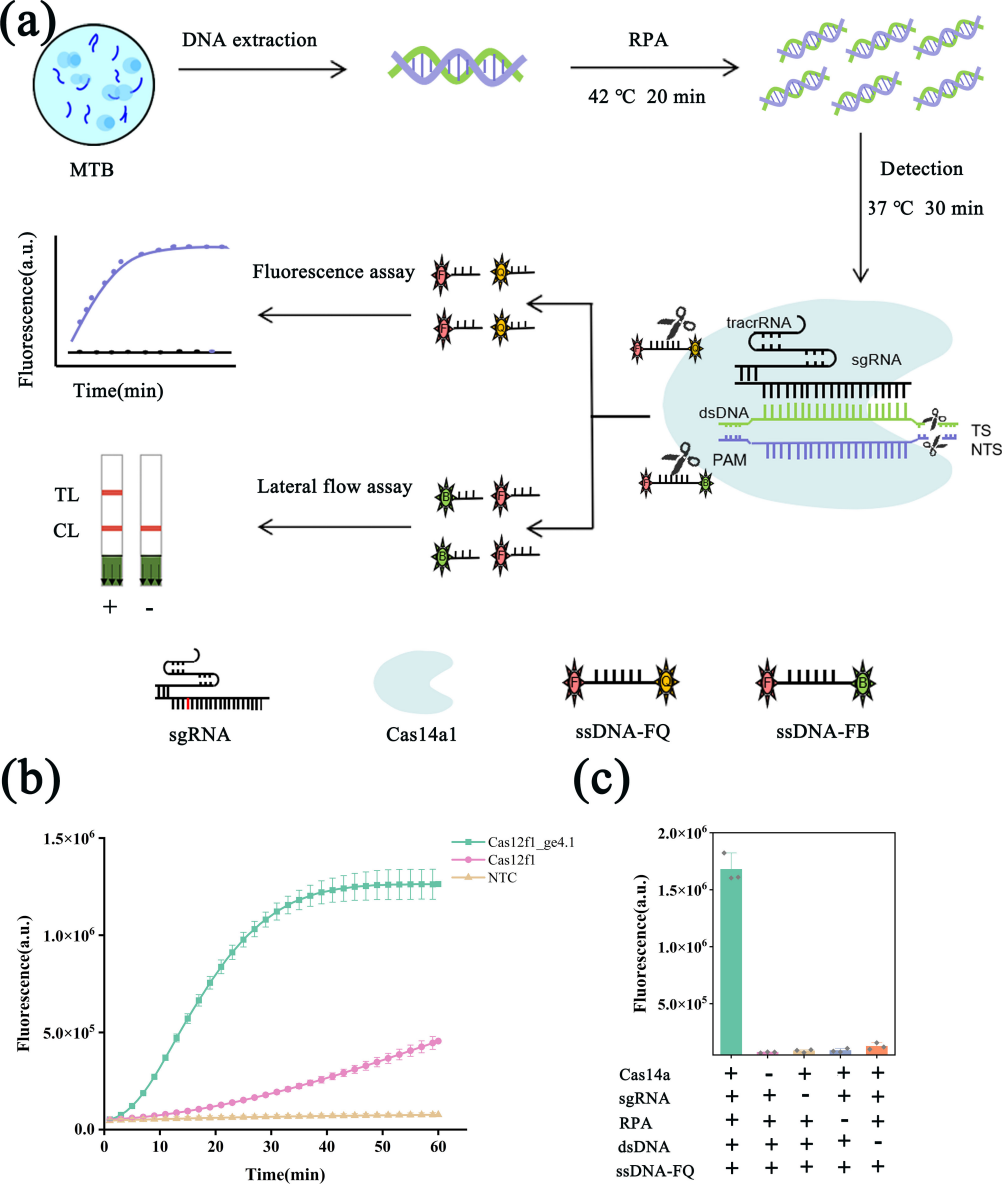
Development of the RPA/CRISPR-Cas12f1_ge4.1 dual-mode detection system. (**a**) Schematic illustration of the working principles of the RPA/Cas12f1_ge4.1 dual-mode reporting system. (**b**) Comparative analysis of detection efficiency between the engineered Cas12f1_ge4.1 system and the wild-type Cas12f1 system. (**c**) Feasibility assessment of the Cas12f1_ge4.1 engineered system for *M. tuberculosis* detection. “+” indicates presence and “-” indicates absence of the component in the system. Error bars represent mean ± SD (*n* = 3).

We developed a novel RPA/CRISPR-Cas12f1_ge4.1 dual-mode system for the rapid and early detection of *M. tuberculosis* DNA. This innovative system integrates the engineered Cas12f1_ge4.1 with recombinase polymerase amplification (RPA), utilizing both fluorescence and lateral flow probes to provide comprehensive detection capabilities. The system operates through three sequential steps: RPA amplification, CRISPR/Cas12f_ge4.1-mediated trans-cleavage, and signal detection (Fig. 1a). In the initial phase, the target sequence (MTB IS6110 or IS1081) undergoes RPA amplification at 37°C–42°C for 20 min. Subsequently, single-guide RNA (sgRNA) forms a complex with the Cas12f1 protein, which recognizes and binds to the double-stranded DNA target via the protospacer adjacent motif (PAM). This interaction activates Cas12f1’s endonuclease activity, leading to target sequence cleavage and collateral cleavage of nearby probes. The resulting fluorescence signals are detectable using a portable fluorescence detector or visible on lateral flow strips.

Building on our previous PDTCTR system, we demonstrate that the engineered Cas12f1_ge4.1 significantly improves detection efficiency for *M. tuberculosis* compared with wild-type Cas12f1 (Fig. 1b; Table S3). To assess system feasibility, we conducted direct detection assays of *M. tuberculosis* DNA. In the presence of *M. tuberculosis* genomic DNA, RPA, and Cas12f1_ge4.1, we observed a strong fluorescence signal plateauing at 30 min. Importantly, the absence of any component resulted in no detectable signal (Fig. 1c). These results confirm that CRISPR-Cas12f1_ge4.1 exhibits collateral cleavage activity triggered by double-stranded DNA targets and, when combined with RPA, enables direct detection of *M. tuberculosis* double-stranded DNA. The dual-mode nature of our system, incorporating both fluorescence and lateral flow detection, offers flexibility for various diagnostic settings, potentially enhancing the accessibility and efficiency of *M. tuberculosis* detection in clinical applications.

### Optimization of the RPA/CRISPR-Cas12f1_ge4.1 fluorescence detection system

**Fig 2:**
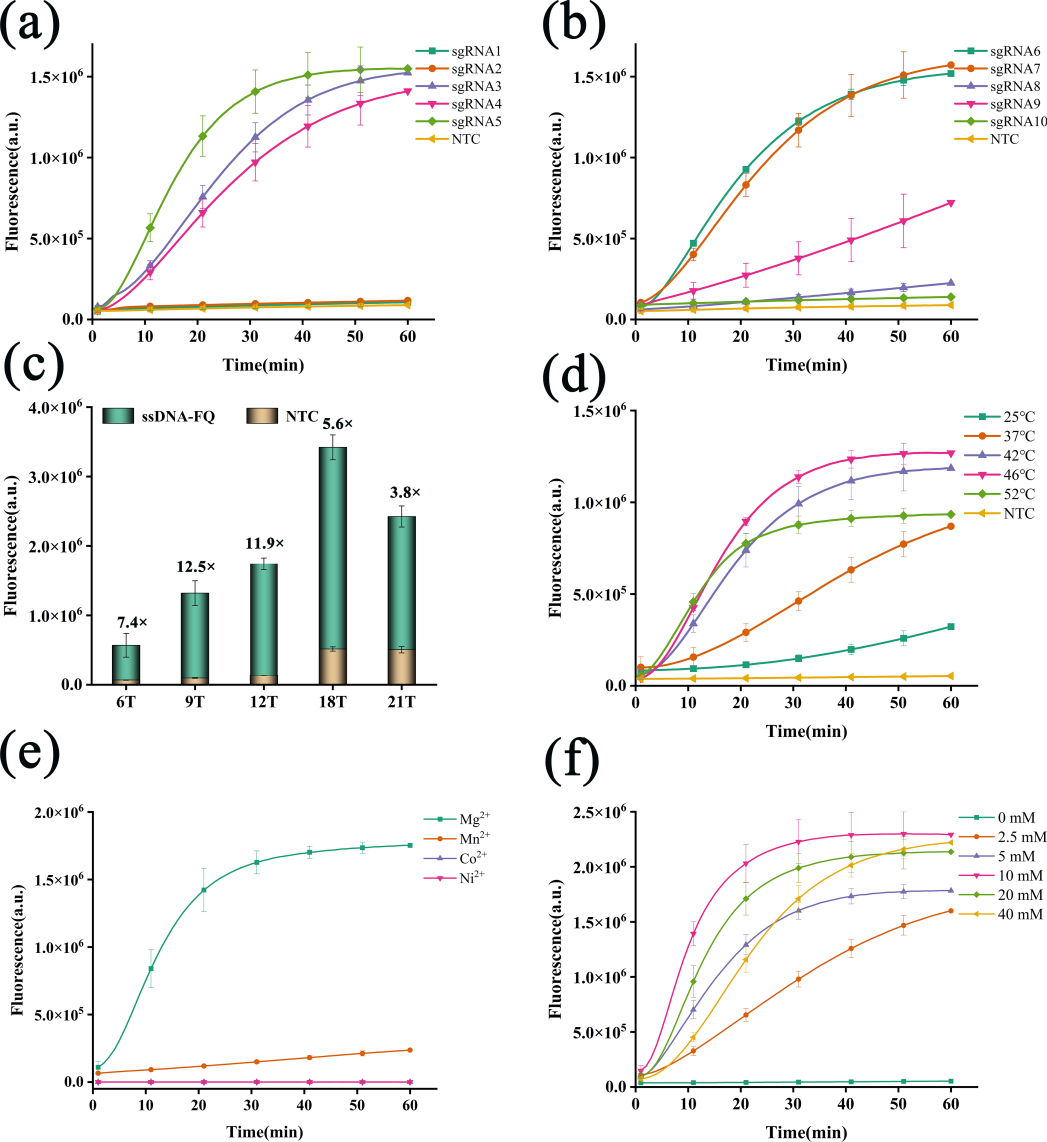
Optimization of the RPA/CRISPR-Cas12f1_ge4.1 fluorescence detection system. (**a**) Selection of optimal sgRNA targeting the MTB-IS6110 gene. (**b**) Selection of optimal sgRNA targeting the MTB-IS1081 gene. (**c**) Optimization of ssDNA-FQ probe length. (**d**) Determination of optimal cleavage temperature. (**e**) Evaluation of divalent metal ion effects. (**f**) Optimization of Mg^2+^ concentration. Error bars represent mean ± SD (*n* = 3 independent replicates).

We conducted a systematic optimization of the RPA/CRISPR-Cas12f1_ge4.1 fluorescence detection system to maximize its performance for *M. tuberculosis* detection. Our comprehensive optimization strategy encompassed key parameters, including target genes, single-guide RNAs (sgRNAs), Cas12f1 protein concentration, fluorescent probes, and reaction conditions. Initially, we screened sgRNAs targeting *M. tuberculosis*-specific insertion sequences IS6110 and IS1081. Among the candidates, IS6110 sgRNA5 and IS1081 sgRNA6 exhibited the highest fluorescence signal intensities (Fig. 2a and b) and were subsequently selected as optimal sgRNAs. Concurrently, we identified IS6110 F1R1 and IS1081 F2R2 as the most effective RPA primer pairs based on fluorescence intensity analysis (Fig. S1a and b).

We then optimized sgRNA concentrations through fluorescence kinetic curve analysis (Fig. S2a and b). IS6110 sgRNA5 demonstrated optimal performance at 150 nM, achieving rapid fluorescence increase and signal saturation within approximately 30 min. IS1081 sgRNA6 showed peak performance at 100 nM. These findings guided the optimization of Cas12f1 protein concentration, which was determined to be optimal at 100 nM (Fig. S3a and b). Further optimization focused on fluorescent probe design and reaction conditions. Balancing the rapid emergence of the fluorescence amplification curve plateau with cost-effectiveness revealed that 12-nucleotide F–Q probes at a concentration of 200 nM provided the best signal-to-noise ratio (Fig. 2c; Fig. S4a and b; Table S4). The system’s highest cleavage efficiency was observed at 46°C (Fig. 2d), with an optimal Mg²^+^ concentration of 10 mM (Fig. 2e and f).

These optimized parameters significantly enhanced the system’s sensitivity for *M. tuberculosis* detection, potentially improving diagnostic capabilities in clinical settings. Our systematic approach provides a robust foundation for developing highly sensitive and specific nucleic acid detection methods for *M. tuberculosis* and potentially other pathogens.

### Development and optimization of the RPA/CRISPR-Cas12f1_ge4.1 lateral flow system

**Fig 3:**
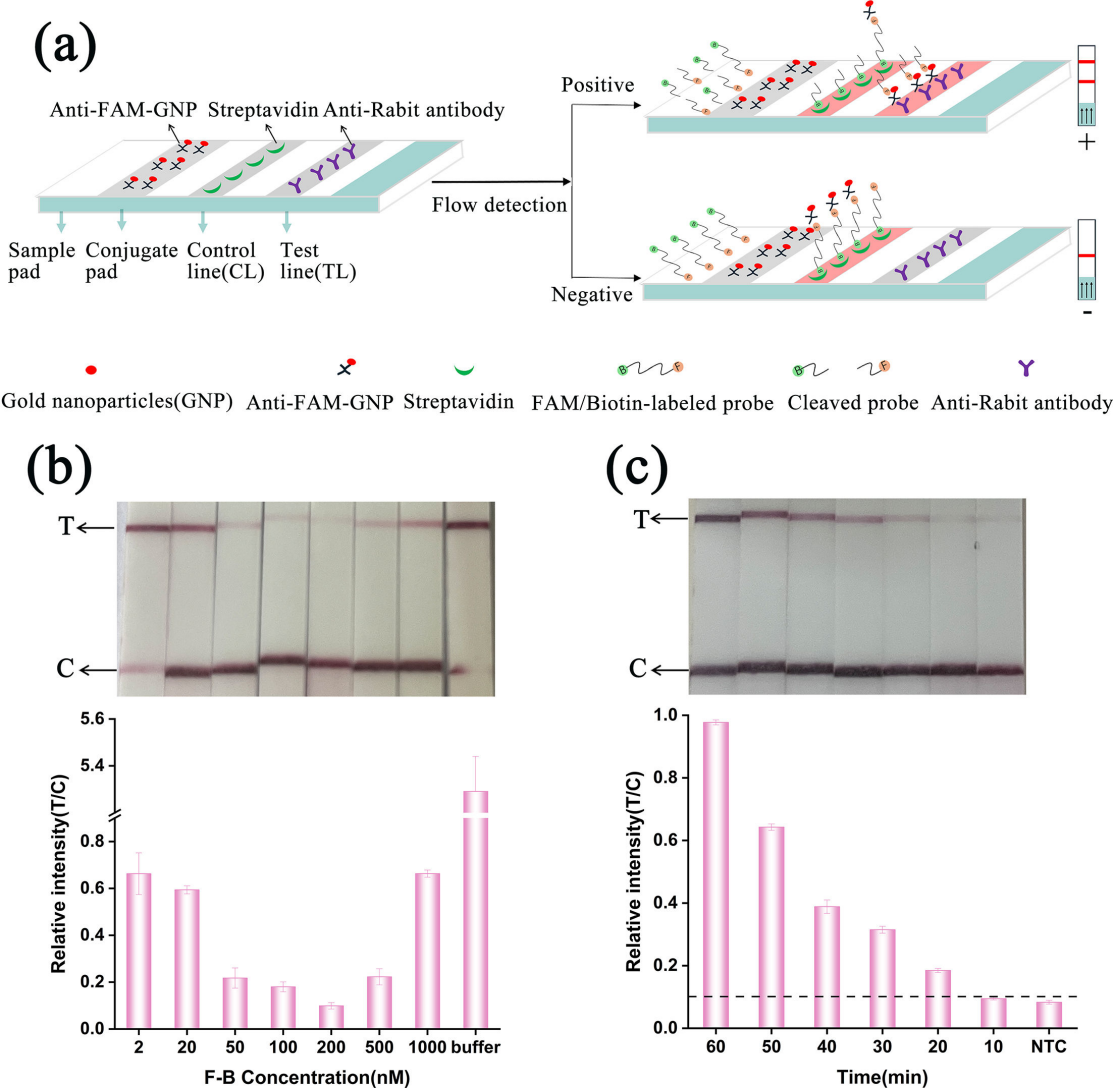
Development and optimization of the RPA/CRISPR-Cas12f1_ge4.1 lateral flow system. (**a**) Schematic illustration of the RPA/Cas12f1_ge4.1 lateral flow system. (**b**) Optimization of F-B reporter molecule concentration for the lateral flow assay. (**c**) Determination of optimal incubation time for the lateral flow assay. LFA strip results for different reaction times are shown at the top, with relative quantifications of band intensities below. NTC: negative control (no template); T: test line; C: control line. Error bars represent mean ± SD (*n* = 3 independent replicates).

To enhance field applicability and reduce reliance on specialized equipment, we developed a novel colloidal gold lateral flow assay integrated with the engineered CRISPR-Cas12f1 system. This RPA/CRISPR-Cas12f1_ge4.1 lateral flow system combines RPA isothermal amplification, engineered Cas12f1 activity, and lateral flow chromatography for visual detection of *M. tuberculosis*. The assay utilizes FAM-Biotin dual-labeled F-B probes and a test strip containing anti-FAM gold-labeled antibodies, streptavidin, and anti-FAM secondary antibodies (Fig. 3a). For the RPA/Cas12f1_ge4.1 lateral flow system, CRISPR-Cas12f1 cleavage products were applied to the sample pad, followed by the running buffer. The running buffer traverses the biosensor via capillary action, rehydrating the coupling pad’s indicator reagents (SA-GNPs). In negative samples, the Cas12f1/sgRNA complex fails to recognize the target DNA, resulting in no collateral cleavage activity. Consequently, the dual-labeled reporter remains intact, becomes bound by mobile anti-FAM antibodies conjugated to GNPS (via FAM at the 5′ end), and is captured by streptavidin immobilized on the control line (CL) (via biotin at the 3′ end). This manifests as a strong control line (C-line) without a test line (T-line). In contrast, in the positive sample, recognition of target DNA by the Cas12f1/sgRNA complex unleashes collateral activity, and subsequently, activated Cas12f1 protein cleaves the dual-labeled reporter, which leads to separation of biotin and FAM labels. The FAM labels are bound by mobile anti-FAM antibodies conjugated to GNPS, and it is then captured by anti-Rabbit antibody immobilized on the TL, and then TL is visualized. Therefore, the presence of target DNA indicates a reported positive result. This design offers significant advantages in terms of rapidity, simplicity, and specificity, particularly for tuberculosis detection in resource-limited settings.

We systematically optimized the system by fine-tuning the F-B reporter molecule concentration. Through iterative testing, we identified 200 nM as the optimal concentration. This concentration effectively eliminated false signals on the T-line in negative samples while maintaining clear differentiation between positive and negative outcomes (Fig. 3b). Quantitative grayscale analysis corroborated these findings, confirming that 200 nM achieves the lowest T/C ratio, thereby ensuring precise result interpretation.

To further optimize the assay, we assessed various incubation times ranging from 10 to 60 min, in 10 min increments, with RPA templates maintained at 46°C. A visible signal emerged within 20 min, with the T/C ratio exceeding the established threshold (defined as the negative control T/C ratio plus three standard deviations) after this duration (Fig. 3c). Consequently, we determined a 20 min incubation period to be optimal, providing a balance between speed and sensitivity for the dipstick reactions.

This optimized lateral flow system offers significant advantages in terms of rapidity, simplicity, and specificity, making it particularly well-suited for tuberculosis detection in resource-limited settings. By eliminating the need for real-time fluorescence detection and allowing direct visual interpretation of results, our system presents a practical, scalable diagnostic solution for point-of-care applications in tuberculosis diagnosis within resource-constrained environments.

### Analytical sensitivity of the RPA/Cas12f1_ge4.1 dual-mode reporting system

**Fig 4:**
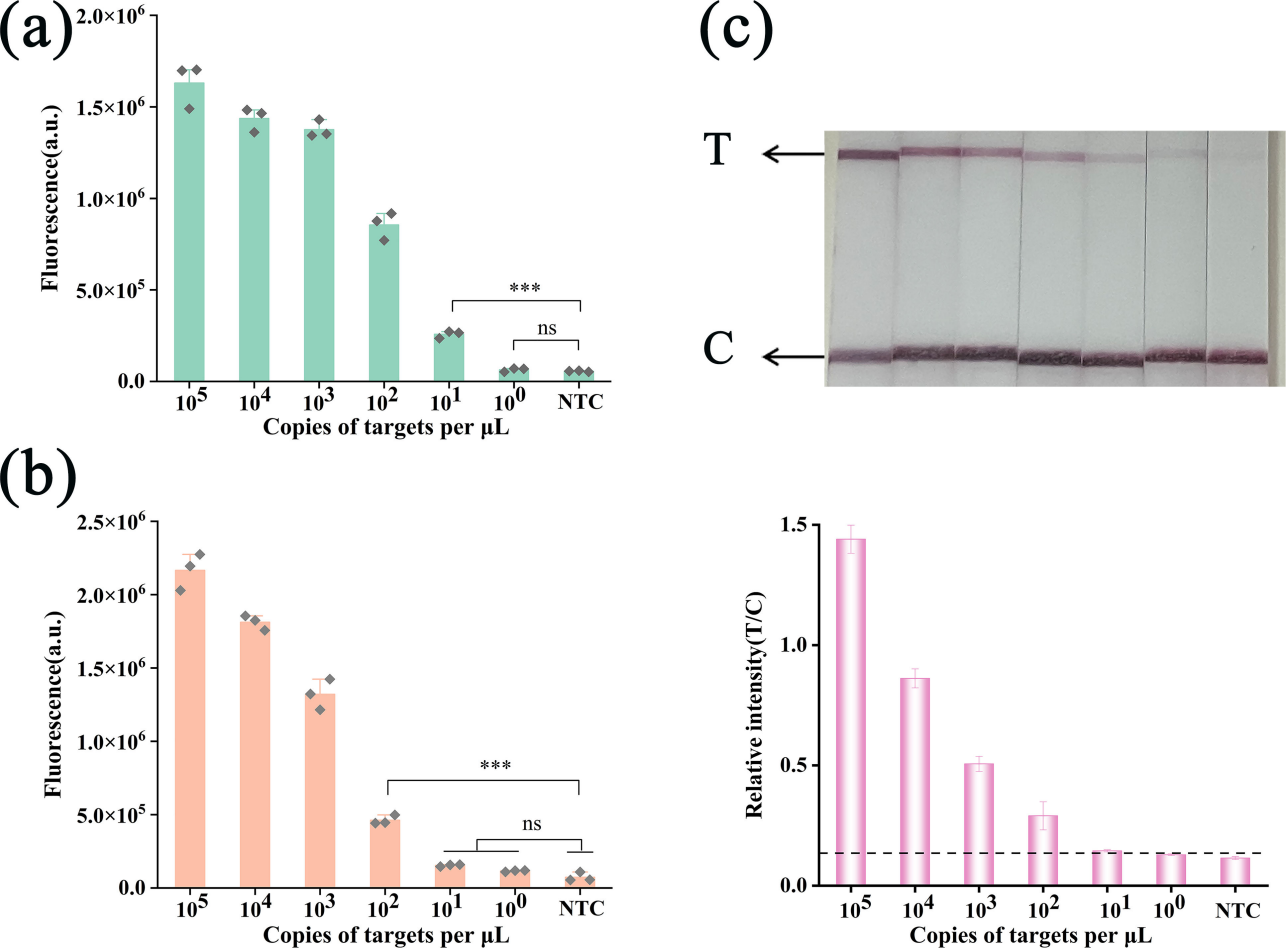
Analytical sensitivity of the RPA/Cas12f1_ge4.1 dual-mode reporting system. (**a**) Sensitivity analysis of the fluorescence system targeting the MTB-IS6110 gene. (**b**) Sensitivity analysis of the fluorescence system targeting the MTB-IS1081 gene. (**c**) Sensitivity validation of the lateral flow system for *M. tuberculosis* detection. Statistical analysis was performed using Brown-Forsythe and Welch ANOVA with Dunnett’s multiple comparison test. ”ns” indicates no significance (*P* > 0.05); asterisks (*, **, ****) denote significant differences with *P* values as indicated. Error bars represent mean ± SD (*n* = 3 independent replicates).

We evaluated the analytical sensitivity of the RPA/Cas12f1_ge4.1 dual-mode reporting system using serial dilutions of *M. tuberculosis* H37Rv DNA to determine the limit of detection (LOD). Fluorescence assays revealed a concentration-dependent decrease in signal accumulation (Fig. S5a and b). The IS6110-sgRNA5 detection system demonstrated superior sensitivity with an LOD of 10 copies/µL, outperforming IS1081-sgRNA6 by an order of magnitude (Fig. 4a and b). Consequently, we selected IS6110-sgRNA5 for further experimentation due to its targeting of a specific insertion sequence.

Lateral flow assays (Fig. 4c) exhibited distinct detection bands at DNA template concentrations of 100 copies/µL and above, with T/C ratios exceeding the predetermined threshold. The dual-mode system thus achieved LODs of 10 and 100 copies/µL for fluorescence and lateral flow detection, respectively. These results underscore the system’s capacity to detect low concentrations of *M. tuberculosis* DNA across both modalities.

These findings validate the high sensitivity of the RPA/Cas12f1_ge4.1 system in detecting low concentrations of *M. tuberculosis* DNA using both fluorescence and lateral flow modalities. This dual-mode system offers robust and reliable detection capabilities, highlighting its potential for sensitive and accessible tuberculosis diagnostics, particularly in resource-limited settings.

### Analytical specificity of the RPA/Cas12f1_ge4.1 dual-mode reporting system

**Fig 5:**
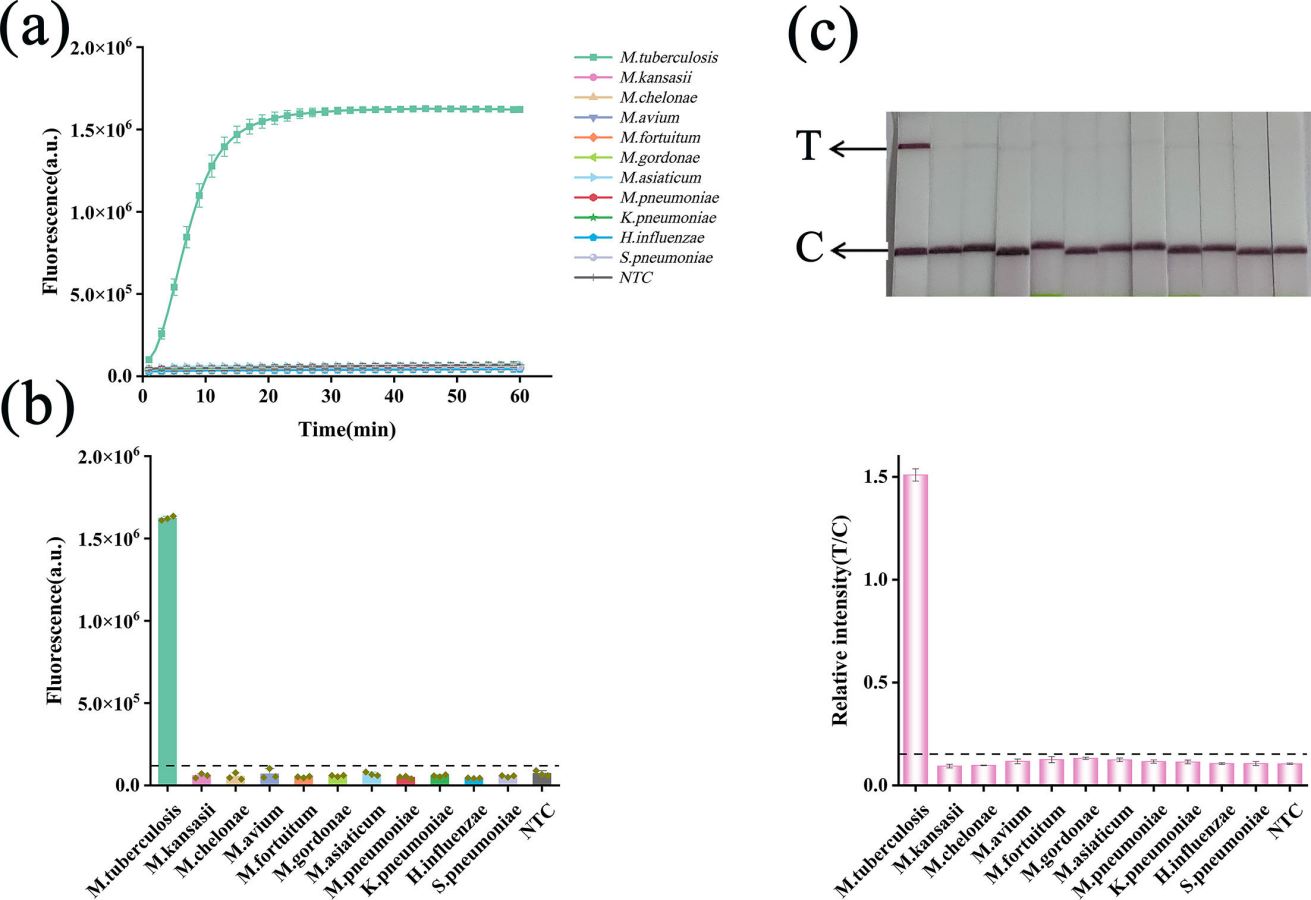
Analytical specificity of the RPA/Cas12f1_ge4.1 dual-mode reporting system. (**a**) Specificity analysis using the fluorescence system. (**b**) Comparison of fluorescence intensities generated by different bacterial templates after 60 min of detection. (**c**) Specificity analysis using the lateral flow system. Error bars represent mean ± SD (*n* = 3 independent replicates).

We assessed the specificity of the RPA/Cas12f1_ge4.1 dual-mode reporting system using genomic DNA from *M. tuberculosis*, non-tuberculous mycobacteria (NTM), and other lung infection-associated bacteria. Fluorescence assays (Fig. 5a and b) demonstrated that only *M. tuberculosis* produced significant fluorescence amplification curves. Lateral flow assays (Fig. 5c) corroborated these results, displaying a distinct detection band exclusively for *M. tuberculosis* templates. Gray-scale scanning analysis further validated these findings, with only the *M. tuberculosis* T/C ratio exceeding the established threshold.

These results demonstrate the high specificity of the RPA/Cas12f1_ge4.1 dual-mode reporting system for *M. tuberculosis* detection. Notably, we observed no cross-reactivity with other mycobacteria or bacterial pathogens. The system’s specificity, coupled with its dual-mode functionality, offers significant advantages for tuberculosis diagnostics, potentially enhancing accuracy and providing versatility in detection methods.

### Clinical application of the RPA/Cas12f1_ge4.1 dual-mode reporting system for *M. tuberculosis* detection

We evaluated the clinical utility of our RPA/Cas12f1_ge4.1 dual-mode reporting system using 113 clinical samples (73 *M*. *tuberculosis* [MTB] positive and 40 negative), previously characterized by qPCR (Table S5). Both fluorescence and lateral flow assays were employed to comprehensively assess the system’s performance across different detection modalities.

Our dual-mode system demonstrated robust performance across both detection formats. The fluorescence-based method achieved exceptional diagnostic accuracy, with a sensitivity of 94.52% (69/73, 95% CI: 85.84%–98.23%) and a specificity of 100% (40/40, 95% CI: 89.09%–100%). Further analysis revealed positive and negative predictive values of 100% and 90.91%, respectively. Moreover, a high kappa coefficient of 0.924 indicated strong concordance with qPCR results. The lateral flow-based system, although slightly less sensitive, also produced impressive outcomes. It demonstrated a sensitivity of 90.41% (66/73, 95% CI: 80.67%–95.73%) and a specificity of 100% (40/40, 95% CI: 89.09%–100%), with positive and negative predictive values of 100% and 85.11%, respectively. A kappa value of 0.878 further confirmed strong concordance with the qPCR results.

**Fig 6:**
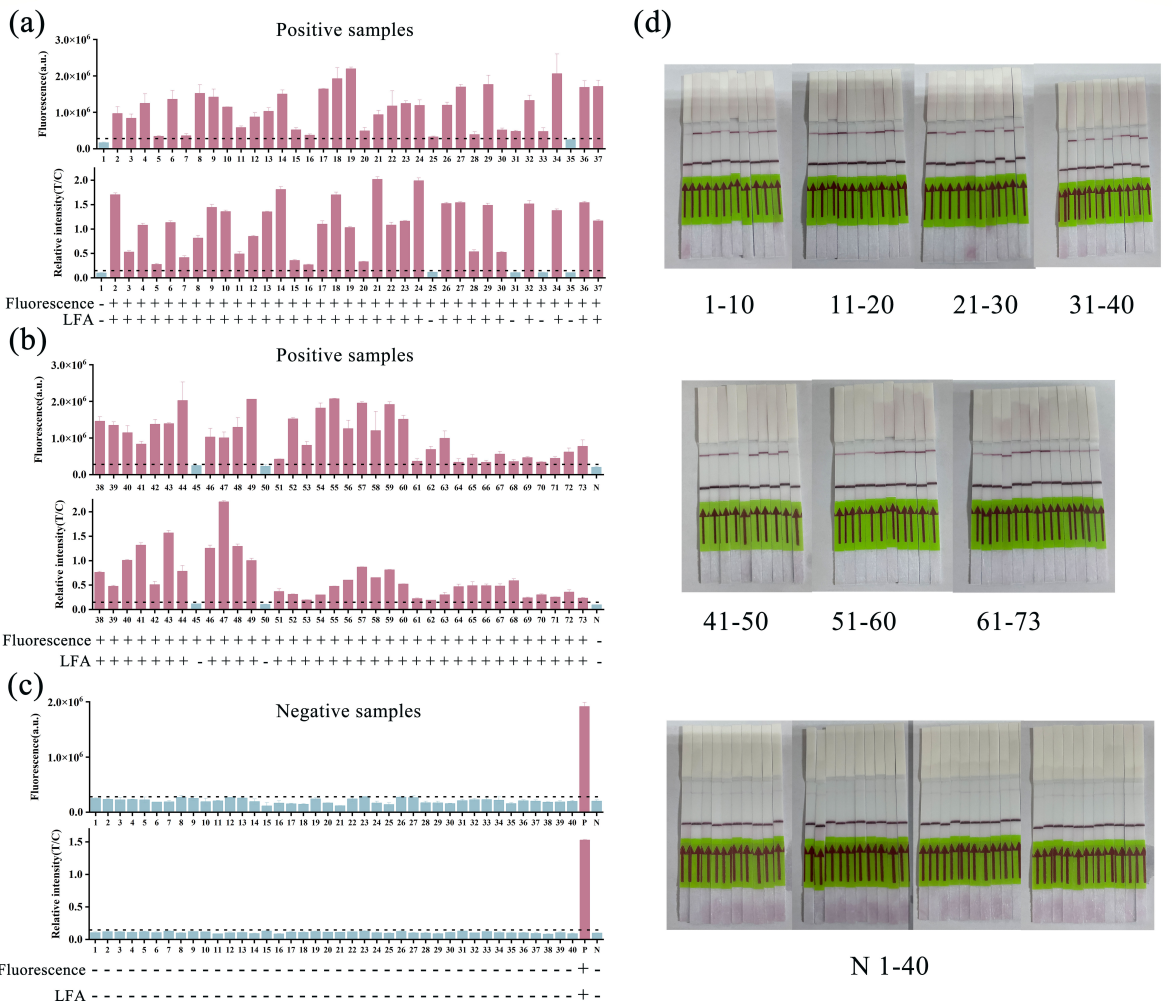
Clinical application of the RPA/Cas12f1_ge4.1 dual-mode reporting system for *M. tuberculosis* detection. (**a–c**) Representative results from the fluorescence assay (top), lateral flow assay (middle), and system interpretation (bottom) for clinical samples. The black horizontal dashed line indicates the threshold for a positive result. +: positive sample; −: negative sample. Error bars represent mean ± SD (*n* = 3 independent replicates). (**d**) Results of the RPA/Cas12f1_ge4.1 lateral flow assay for the detection of 113 *M*. *tuberculosis* clinical samples.

The dual-mode detection capability, encompassing both fluorescence and lateral flow readouts, enables seamless adaptation to diverse clinical settings, from well-equipped laboratories to resource-limited environments (Fig. 6; Table 1).

**TABLE 1:** Comparative analysis of *M. tuberculosis* detection in clinical samples using the RPA/CRISPR-Cas12f1_ge4.1 dual-mode system and qPCR^*a*^

		RPA/CRISPR–Cas12f1_ge4.1 fluorescence system		Total	RPA/CRISPR–Cas12f1_ge4.1 lateral flow system		Total
		+	−		+	−	
qPCR result	+	69	4	73	66	7	73
	−	0	40	40	0	40	40
Total		69	44	113	66	47	113
Sensitivity		94.52%			90.41%		
Specificity		100%			100%		
κ		0.924			0.878		

^*a*^ +: positive; −: negative. Sensitivity, specificity, and kappa values were calculated using SPSS 25.0 software.

## DISCUSSION

The global tuberculosis burden necessitates innovative diagnostic strategies that integrate rapidity, sensitivity, and accessibility (20, 21). We present a novel RPA/Cas12f1_ge4.1 dual-mode reporting system that addresses critical limitations in current *M. tuberculosis* detection methods. Our system demonstrates robust sensitivity, specificity, and versatility, offering significant advancements in tuberculosis diagnostics across diverse clinical settings.

Our RPA/Cas12f1_ge4.1 system achieves a remarkable detection limit of 10 copies/µL for *M. tuberculosis*, comparable to the PDTCTR system and competitive with advanced CRISPR-based diagnostics platforms, such as SHERLOCK (22) and HOLMES (23), while also rivaling Cas12a-based MCMD methods (9). Notably, this system outperforms conventional nucleic acid amplification techniques, including RPA (24) and LAMP (25). The enhanced sensitivity and specificity of our system can be attributed to two critical factors: engineered sgRNA and optimal target selection. Our findings corroborate and extend previous research demonstrating that strategic sgRNA modifications significantly augment Cas12f1 activity (19, 26). Specifically, the removal of the stem-loop 2 region and the introduction of a U-rich 3′ overhang in the sgRNA design have proven instrumental in optimizing the system’s performance. Furthermore, our comparative analysis of IS6110-targeted and IS1081-targeted sgRNAs reveals the paramount importance of target selection in CRISPR-based diagnostics. The superior performance of the IS6110-targeted sgRNA underscores the necessity of careful consideration in choosing genomic targets to maximize diagnostic efficacy.

To enhance point-of-care applicability, we developed a lateral flow adaptation capable of detecting *M. tuberculosis* at 100 copies/µL through visual interpretation, comparable to CRISPR–Cas12a-based methods (27). This advancement eliminates the need for complex instrumentation, making it ideal for resource-limited settings. The rapid turnaround time of 40–60 min facilitates faster treatment initiation and implementation of effective disease control strategies compared with conventional culture-based methods, which can take weeks (28). While fluorescence detection offers high sensitivity and quantitative capabilities, lateral flow detection excels in speed, simplicity, and cost-effectiveness. Fluorescence methods are commonly used in research and clinical laboratories for in-depth analyses, whereas lateral flow assays are well suited for point-of-care testing, home diagnostics, and field applications where prompt results are essential.

Clinical validation using 113 samples demonstrated the system’s robustness, with sensitivity exceeding 90% and 100% specificity. This performance surpasses the WHO-recommended Xpert MTB/RIF assay (85% sensitivity, 98% specificity) (29) and previously reported CRISPR-based assays, including a Cas12a-based assay (79.5% sensitivity, 100% specificity) (30) and a Cas12b-based assay (86.8% sensitivity, 95.3% specificity) (10). These results highlight the reliability and accuracy of both detection methods in our RPA/Cas12f1_ge4.1 dual-mode reporting system.

Despite its strengths, our system presents opportunities for further refinement. The two-step process, while effective, introduces potential contamination risks and extends the overall detection time compared to one-step methods. Future research should prioritize the development of single-step or amplification-free approaches to mitigate these limitations, further streamlining the workflow for broader clinical applicability. While our system demonstrates high sensitivity, further optimization to reach the gold standard of qPCR (31) is warranted. Exploring alternative Cas12f1 variants or employing directed evolution to enhance the protein’s catalytic activity could yield greater sensitivity.

To enhance clinical applicability, future studies should evaluate a broader range of sample types beyond sputum. Given the diagnostic challenges of extrapulmonary tuberculosis, it is essential to assess test performance using samples, such as pleural effusion, cerebrospinal fluid, and circulating cell-free DNA (cfDNA) (32–34). Additionally, larger-scale clinical trials conducted across diverse settings are needed to thoroughly evaluate the system’s real-world performance and robustness. Moreover, integrating the CRISPR platform with various technologies—such as microfluidic chips, droplet microfluidics, electrochemistry, and optical systems—could enable nucleic acid amplification-free detection, thereby reducing the risk of contamination (35, 36).

### Conclusion

In summary, our novel RPA/CRISPR-Cas12f1_ge4.1 dual-mode system offers rapid and sensitive detection of *M. tuberculosis*, demonstrating versatility in both laboratory and resource-limited settings. With high analytical performance and clinical validation, it represents a significant advancement in tuberculosis diagnostics. Future work will focus on optimizing workflow and expanding sample compatibility, potentially revolutionizing global tuberculosis management and contributing to more effective disease control strategies.

## SUPPLEMENTAL MATERIAL

Supplemental figures spectrum.02652-24-s0001.docx Fig. S1 to S5.

Supplemental tables spectrum.02652-24-s0002.docx Tables S1 to S4.

Table S5 spectrum.02652-24-s0003.xlsx Experimental result.

ASM does not own the copyrights to Supplemental Material that may be linked to, or accessed through, an article. The authors have granted ASM a non-exclusive, world-wide license to publish the Supplemental Material files. Please contact the corresponding author directly for reuse.

## References

[1] Ankrah AO, Glaudemans A, Maes A, Van de Wiele C, Dierckx R, Vorster M, Sathekge MM. 2018. Tuberculosis. Semin Nucl Med 48:108–130. doi:10.1053/j.semnuclmed.2017.10.005 29452616

[2] World Health Organization. 2024. Global tuberculosis report 2024. World Health Organization, Geneva.

[3] Fleming KA, Horton S, Wilson ML, Atun R, DeStigter K, Flanigan J, Sayed S, Adam P, Aguilar B, Andronikou S, et al.. 2021. The Lancet commission on diagnostics: transforming access to diagnostics. Lancet 398:1997–2050. doi:10.1016/S0140-6736(21)00673-5 34626542 PMC8494468

[4] Steingart KR, Henry M, Ng V, Hopewell PC, Ramsay A, Cunningham J, Urbanczik R, Perkins M, Aziz MA, Pai M. 2006. Fluorescence versus conventional sputum smear microscopy for tuberculosis: a systematic review. Lancet Infect Dis 6:570–581. doi:10.1016/S1473-3099(06)70578-3 16931408

[5] Machado D, Couto I, Viveiros M. 2019. Advances in the molecular diagnosis of tuberculosis: from probes to genomes. Infect Genet Evol 72:93–112. doi:10.1016/j.meegid.2018.11.021 30508687

[6] Gong X, He Y, Zhou K, Hua Y, Li Y. 2023. Efficacy of Xpert in tuberculosis diagnosis based on various specimens: a systematic review and meta-analysis. Front Cell Infect Microbiol 13:1149741. doi:10.3389/fcimb.2023.1149741 37201118 PMC10185844

[7] Zetsche B, Gootenberg JS, Abudayyeh OO, Slaymaker IM, Makarova KS, Essletzbichler P, Volz SE, Joung J, van der Oost J, Regev A, Koonin EV, Zhang F. 2015. Cpf1 is a single RNA-guided endonuclease of a class 2 CRISPR-Cas system. Cell 163:759–771. doi:10.1016/j.cell.2015.09.038 26422227 PMC4638220

[8] O’Connell MR. 2019. Molecular mechanisms of RNA targeting by Cas13-containing type VI CRISPR–Cas systems. J Mol Biol 431:66–87. doi:10.1016/j.jmb.2018.06.029 29940185

[9] Xiao J, Li J, Quan S, Wang Y, Jiang G, Wang Y, Huang H, Jiao W, Shen A. 2023. Development and preliminary assessment of a CRISPR–Cas12a-based multiplex detection of Mycobacterium tuberculosis complex. Front Bioeng Biotechnol 11:1233353. doi:10.3389/fbioe.2023.1233353 37711452 PMC10497956

[10] Sam IK, Chen YY, Ma J, Li SY, Ying RY, Li LX, Ji P, Wang SJ, Xu J, Bao YJ, Zhao GP, Zheng HJ, Wang J, Sha W, Wang Y. 2021. TB-QUICK: CRISPR-Cas12b-assisted rapid and sensitive detection of Mycobacterium tuberculosis. J Infect 83:54–60. doi:10.1016/j.jinf.2021.04.032 33951419

[11] Ren W, Zhou Y, Li H, Shang Y, Zhang X, Yuan J, Li S, Li C, Pang Y. 2023. Development and clinical evaluation of a CRISPR/Cas13a-based diagnostic test to detect Mycobacterium tuberculosis in clinical specimens. Front Microbiol 14:1117085. doi:10.3389/fmicb.2023.1117085 36819015 PMC9935578

[12] Harrington LB, Burstein D, Chen JS, Paez-Espino D, Ma E, Witte IP, Cofsky JC, Kyrpides NC, Banfield JF, Doudna JA. 2018. Programmed DNA destruction by miniature CRISPR-Cas14 enzymes. Science 362:839–842. doi:10.1126/science.aav4294 30337455 PMC6659742

[13] Ai JW, Zhou X, Xu T, Yang M, Chen Y, He GQ, Pan N, Cai Y, Li Y, Wang X, Su H, Wang T, Zeng W, Zhang WH. 2019. CRISPR-based rapid and ultra-sensitive diagnostic test for Mycobacterium tuberculosis. Emerg Microbes Infect 8:1361–1369. doi:10.1080/22221751.2019.1664939 31522608 PMC6758691

[14] Xu X, Chemparathy A, Zeng L, Kempton HR, Shang S, Nakamura M, Qi LS. 2021. Engineered miniature CRISPR-Cas system for mammalian genome regulation and editing. Mol Cell 81:4333–4345. doi:10.1016/j.molcel.2021.08.008 34480847

[15] Wang Y, Wang Y, Pan D, Yu H, Zhang Y, Chen W, Li F, Wu Z, Ji Q. 2022. Guide RNA engineering enables efficient CRISPR editing with a miniature Syntrophomonas palmitatica Cas12f1 nuclease. Cell Rep 40:111418. doi:10.1016/j.celrep.2022.111418 36170834

[16] Kim DY, Lee JM, Moon SB, Chin HJ, Park S, Lim Y, Kim D, Koo T, Ko JH, Kim YS. 2022. Efficient CRISPR editing with a hypercompact Cas12f1 and engineered guide RNAs delivered by adeno-associated virus. Nat Biotechnol 40:94–102. doi:10.1038/s41587-021-01009-z 34475560 PMC8763643

[17] Chen JS, Ma E, Harrington LB, Da Costa M, Tian X, Palefsky JM, Doudna JA. 2018. CRISPR-Cas12a target binding unleashes indiscriminate single-stranded DNase activity. Science 360:436–439. doi:10.1126/science.aar6245 29449511 PMC6628903

[18] Wei Y, Yang Z, Zong C, Wang B, Ge X, Tan X, Liu X, Tao Z, Wang P, Ma C, Wan Y, Li J. 2021. trans single-stranded DNA cleavage via CRISPR/Cas14a1 activated by target RNA without destruction. Angew Chem Int Ed Engl 60:24241–24247. doi:10.1002/anie.202110384 34553468

[19] He J, Hu X, Weng X, Wang H, Yu J, Jiang T, Zou L, Zhou X, Lyu Z, Liu J, Zhou P, Xiao X, Zhen D, Deng Z. 2024. Efficient, specific and direct detection of double-stranded DNA targets using Cas12f1 nucleases and engineered guide RNAs. Biosens Bioelectron 260:116428. doi:10.1016/j.bios.2024.116428 38805891

[20] Reid M, Agbassi YJP, Arinaminpathy N, Bercasio A, Bhargava A, Bhargava M, Bloom A, Cattamanchi A, Chaisson R, Chin D, et al.. 2023. Scientific advances and the end of tuberculosis: a report from the Lancet Commission on Tuberculosis. Lancet 402:1473–1498. doi:10.1016/S0140-6736(23)01379-X 37716363

[21] Houben R, Dodd PJ. 2016. The global burden of Latent tuberculosis infection: a re-estimation using mathematical modelling. PLoS Med 13:e1002152. doi:10.1371/journal.pmed.1002152 27780211 PMC5079585

[22] Gootenberg JS, Abudayyeh OO, Lee JW, Essletzbichler P, Dy AJ, Joung J, Verdine V, Donghia N, Daringer NM, Freije CA, Myhrvold C, Bhattacharyya RP, Livny J, Regev A, Koonin EV, Hung DT, Sabeti PC, Collins JJ, Zhang F. 2017. Nucleic acid detection with CRISPR-Cas13a/C2c2. Science 356:438–442. doi:10.1126/science.aam9321 28408723 PMC5526198

[23] Li SY, Cheng QX, Wang JM, Li XY, Zhang ZL, Gao S, Cao RB, Zhao GP, Wang J. 2018. CRISPR-Cas12a-assisted nucleic acid detection. Cell Discov 4:20. doi:10.1038/s41421-018-0028-z 29707234 PMC5913299

[24] Sciaudone M, Carpena R, Calderón M, Sheen P, Zimic M, Coronel J, Gilman RH, Bowman NM. 2023. Rapid detection of Mycobacterium tuberculosis using recombinase polymerase amplification: a pilot study. PLoS One 18:e0295610. doi:10.1371/journal.pone.0295610 38064441 PMC10707601

[25] Yadav R, Daroch P, Gupta P, Vaidya P, Mathew JL, Singh M, Sethi S. 2021. Evaluation of TB-LAMP assay for detection of Mycobacterium tuberculosis in children. Infect Dis (Lond) 53:942–946. doi:10.1080/23744235.2021.1963467 34403308

[26] Jiang T, Wei G, Lin M, Zhang S, Zou L, Zhou X, Deng Z. 2025. A ERA/Cas12f1_ge4.1 biosensor for rapid, sensitive, and cost-effective detection of Chlamydia psittaci via fluorescence and lateral flow assays. Talanta 287:127615. doi:10.1016/j.talanta.2025.127615 39862519

[27] Deng Z, Hu H, Tang D, Liang J, Su X, Jiang T, Hu X, Ying W, Zhen D, Xiao X, He J. 2022. Ultrasensitive, specific, and rapid detection of Mycoplasma pneumoniae using the ERA/CRISPR–Cas12a dual system. Front Microbiol 13:811768. doi:10.3389/fmicb.2022.811768 35633705 PMC9136402

[28] Asmar S, Drancourt M. 2015. Rapid culture-based diagnosis of pulmonary tuberculosis in developed and developing countries. Front Microbiol 6:1184. doi:10.3389/fmicb.2015.01184 26579092 PMC4630581

[29] Pillay S, Steingart KR, Davies GR, Chaplin M, De Vos M, Schumacher SG, Warren R, Theron G. 2022. Xpert MTB/XDR for detection of pulmonary tuberculosis and resistance to isoniazid, fluoroquinolones, ethionamide, and amikacin. Cochrane Database Syst Rev 5:CD014841. doi:10.1002/14651858.CD014841.pub2 35583175 PMC9115865

[30] Wang Y, Li J, Li S, Zhu X, Wang X, Huang J, Yang X, Tai J. 2021. LAMP-CRISPR-Cas12-based diagnostic platform for detection of Mycobacterium tuberculosis complex using real-time fluorescence or lateral flow test. Microchim Acta 188:347. doi:10.1007/s00604-021-04985-w 34542728

[31] Reed JL, Walker ZJ, Basu D, Allen V, Nicol MP, Kelso DM, McFall SM. 2016. Highly sensitive sequence specific qPCR detection of Mycobacterium tuberculosis complex in respiratory specimens. Tuberculosis (Edinb) 101:114–124. doi:10.1016/j.tube.2016.09.002 27865380

[32] Svensson E, Folkvardsen DB, Rasmussen EM, Lillebaek T. 2021. Detection of Mycobacterium tuberculosis complex in pulmonary and extrapulmonary samples with the FluoroType MTBDR assay. Clin Microbiol Infect 27:1514. doi:10.1016/j.cmi.2020.12.020 33421581

[33] Liu Y, Liu S, Zhen D, Huang J, He F. 2024. Ultrasensitive detection of tumor suppressor gene methylation by piezoelectric sensing based on enrichment of transcription activator-like effectors. Anal Chem 96:8534–8542. doi:10.1021/acs.analchem.4c00484 38743638

[34] Thakku SG, Lirette J, Murugesan K, Chen J, Theron G, Banaei N, Blainey PC, Gomez J, Wong SY, Hung DT. 2023. Genome-wide tiled detection of circulating Mycobacterium tuberculosis cell-free DNA using Cas13. Nat Commun 14:1803. doi:10.1038/s41467-023-37183-8 37002219 PMC10064635

[35] Zhen D, Zhang S, Yang A, Ma Q, Deng Z, Fang J, Cai Q, He J. 2024. A supersensitive electrochemical sensor based on RCA amplification-assisted "silver chain"-linked gold interdigital electrodes and CRISPR/Cas9 for the detection of Staphylococcus aureus in food. Food Chem 440:138197. doi:10.1016/j.foodchem.2023.138197 38104453

[36] Qian S, Chen Y, Xu X, Peng C, Wang X, Wu H, Liu Y, Zhong X, Xu J, Wu J. 2022. Advances in amplification-free detection of nucleic acid: CRISPR/Cas system as a powerful tool. Anal Biochem 643:114593. doi:10.1016/j.ab.2022.114593 35157895

