# Supplementary material for: Rapid and sensitive detection of Mycobacterium tuberculosis using the RPA/Cas12f1_ge4.1 system with fluorescence and lateral flow readouts
Source: Microbiol Spectr. 2025 Jun 9;13(7):e02652-24. doi: 10.1128/spectrum.02652-24 (PMC12211024; doi:10.1128/spectrum.02652-24)
Supplement: Supplemental figures — Fig. S1 to S5. [file spectrum.02652-24-s0001.docx]

**Rapid and Sensitive Detection of *Mycobacterium tuberculosis* Using the RPA/Cas12f1_ge4.1 System with Fluorescence and Lateral Flow Readouts**

Zhongliang Deng^1,2, †^, Xingyong Weng^2, 3, †^, Honghua Tang^4, †^, Tintao Zou^2^, Xuan Zhou^2^, Hangxi Liu^2^, Piaoting Wen^2^, Gemiao Luo^2^, Tian Gan^1, *^, Jun He^1, *^


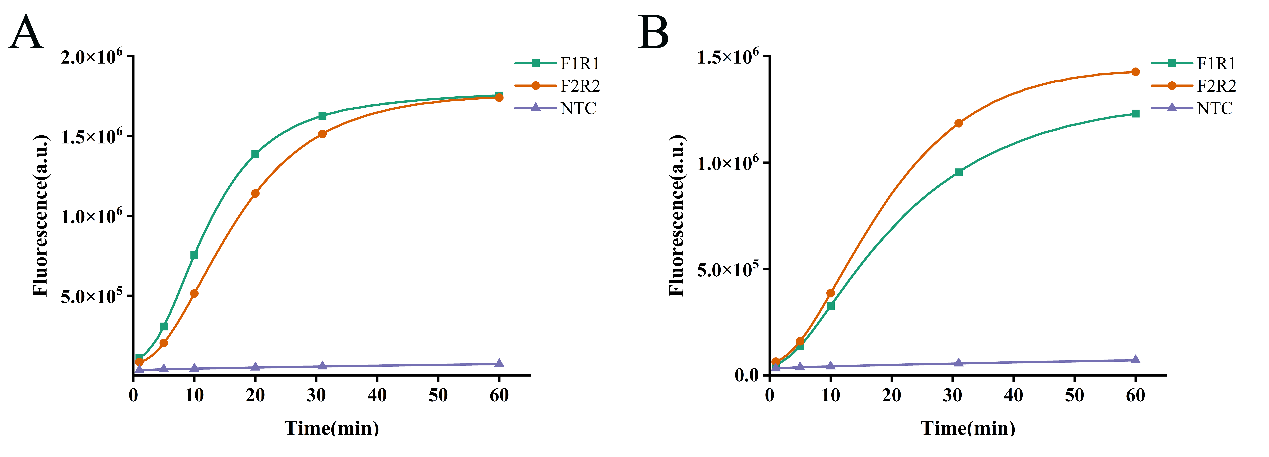


**Supplementary Figure S1 Optimization of RPA primer in the RPA/Cas12f1_ge4.1 fluorescence system. (**A) Optimization of RPA primers targeting the MTB-IS6110 gene. (B) Optimization of RPA primers targeting the MTB-IS1081 gene.


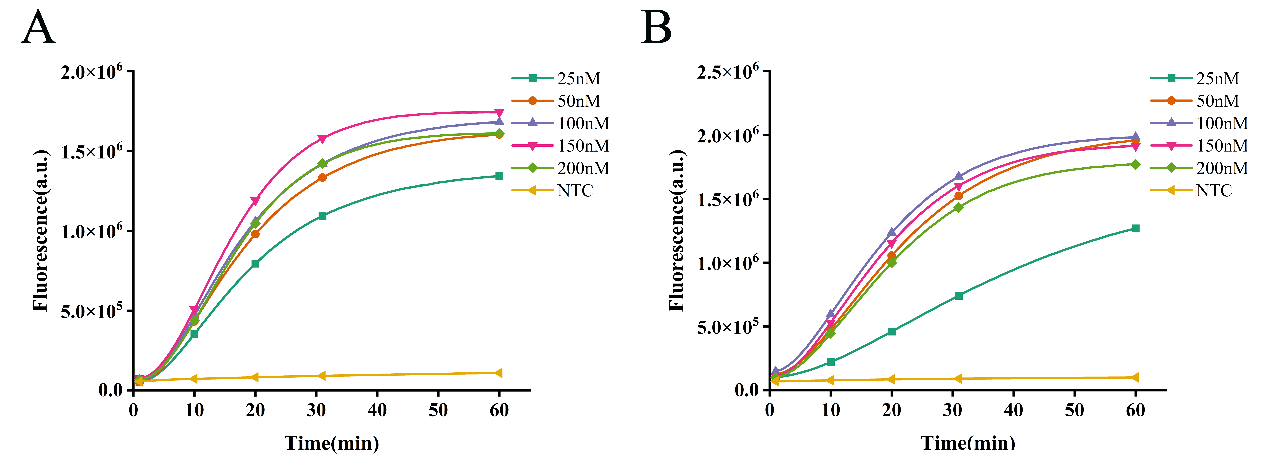


**Supplementary Figure S2 Optimization of sgRNA concentration in the RPA/Cas12f1_ge4.1 fluorescence system.** (A) Optimization of sgRNA concentration targeting the MTB-IS6110 gene. (B) Optimization of sgRNA concentration targeting the MTB-IS1081 gene.


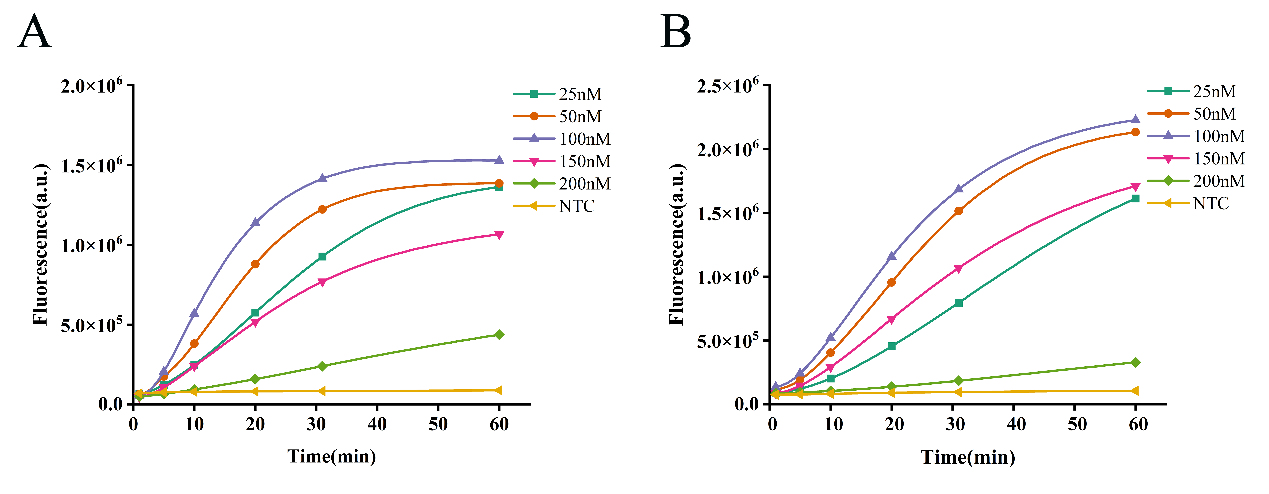


**Supplementary Figure S3 Optimization of Cas12f1 concentration in the RPA/Cas12f1_ge4.1 fluorescence system.** (A) Optimization of Cas12f1 concentration targeting the MTB-IS6110 gene. (B) Optimization of Cas12f1 concentration targeting the MTB-IS1081 gene.


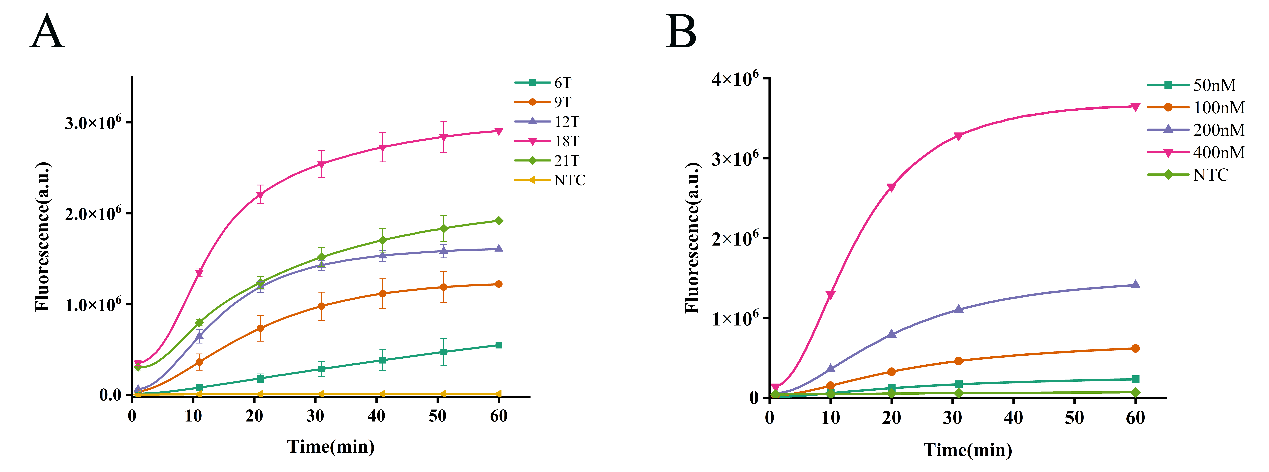


**Supplementary Figure S4 Optimization of ssDNA-FQ in theRPA/Cas12f1_ge4.1 fluorescence system.** (A) Optimization of ssDNA-FQ length (B) Optimization of ssDNA-FQ concentration.


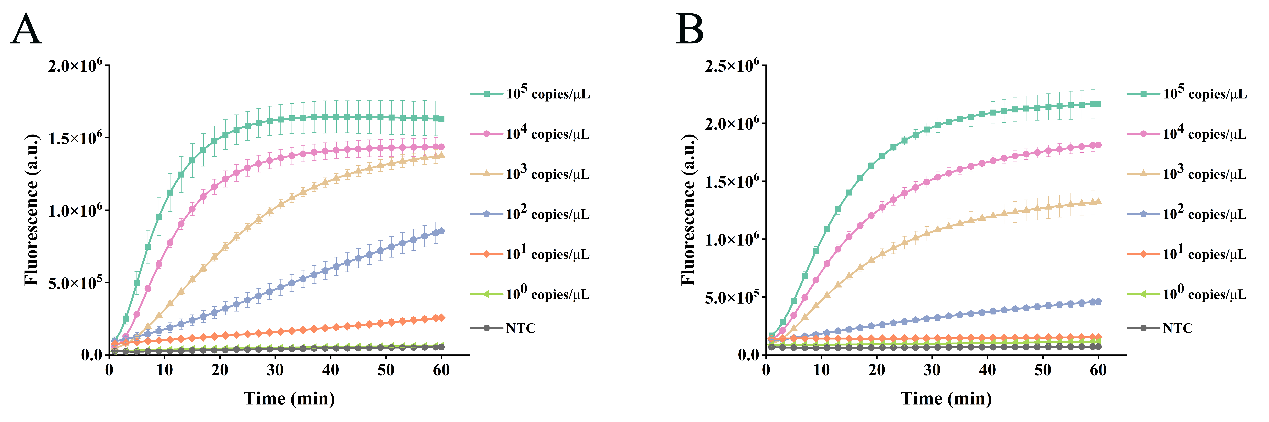


**Supplementary Figure S5 Analytical sensitivity of the RPA/Cas12f1_ge4.1 fluorescence system.** (A) Sensitivity analysis of the RPA/Cas12f1_ge4.1 fluorescence system for detecting the MTB-IS6110 target gene (B) Sensitivity analysis of the RPA/Cas12f1_ge4.1 fluorescence system for detecting the MTB-IS1081 target gene.
