# Supplementary material for: Rapid and sensitive detection of Mycobacterium tuberculosis using the RPA/Cas12f1_ge4.1 system with fluorescence and lateral flow readouts
Source: Microbiol Spectr. 2025 Jun 9;13(7):e02652-24. doi: 10.1128/spectrum.02652-24 (PMC12211024; doi:10.1128/spectrum.02652-24)
Supplement: Supplemental tables — Tables S1 to S4. [file spectrum.02652-24-s0002.docx]

**Supplementary Table S1. The sequence of RPA Primers**

| Name | Gene | RPA Primer | Sequence (5’-3’) | Length(bp) |
| --- | --- | --- | --- | --- |
| MTB | IS6100 | sgRNA1-F | GAATTGCGAAGGGCGAACGCGATTTTAAAG | 30 |
|  |  | sgRNA1-R | GGGCTCCCGGTTGATGTGGTCGTAGTAGGT | 30 |
|  |  | sgRNA2-F | CTGTGGGTAGCAGACCTCACCTATGTGTCG | 30 |
|  |  | sgRNA2-R | TGAGATCCCCTATCCGTATGGTGGATAACG | 30 |
|  |  | sgRNA3-F | GGTCGGAAGCTCCTATGACAATGCACTAGCC | 31 |
|  |  | sgRNA3-R | TTGAGCGTAGTAGGCAGCCTCGAGTTCGAC | 30 |
|  |  | sgRNA4-F | TGGTTTGCGGTGGGGTGTCGAGTCGATCTG | 30 |
|  |  | sgRNA4-R | GTGGTCCCGGACAGGCCGAGTTTGGTCATC | 30 |
|  |  | sgRNA5-F1 | CAACTACGGTGTTTACGGTGCCCGCAAAGTG | 31 |
|  |  | sgRNA5-R1 | AGGTGAGGTCTGCTACCCACAGCCGGTTAGG | 31 |
|  |  | sgRNA5-F2 | CACGCCGCCAACTACGGTGTTTACGGTGCC | 30 |
|  |  | sgRNA5-R2 | GGATCAGCGATCGTGGTCCTGCGGGCTTTG | 30 |
|  | IS1081 | sgRNA6-F1 | GAATTCGATCGCCGAGCCGACAAGACATGC | 30 |
|  |  | sgRNA6-R1 | ACACCCCTGGACGTAACTTGACACCAATCC | 30 |
|  |  | sgRNA6-F2 | CGACAAGACATGCCAGCGCAACCCGCTTCA | 30 |
|  |  | sgRNA6-R2 | ACGCCCACCGGCCTTACCTGCCCGTACACC | 30 |
|  |  | sgRNA7-F | CTGCTGGGAGTATCCACTCGCCGGATGGAGC | 31 |
|  |  | sgRNA7-R | GTCCGAAACGCCTCTACGGCTTCGTCGAGCT | 31 |
|  |  | sgRNA8-F | CAATCTGATGGCAGCCACCCCGAAGCCCTCC | 31 |
|  |  | sgRNA8-R | CAGGTCGGTGCGGGCGGTGTCGAGGTGCTC | 30 |
|  |  | sgRNA9-F | CCAAGCTGCGCCAGGGCAGCTATTTCCCGGAC | 32 |
|  |  | sgRNA9-R | TTGGCCATGATCGACACTTGCGACTTGGA | 29 |
|  |  | sgRNA10-F | GAATTCGATCGCCGAGCCGACAAGACATGC | 30 |
|  |  | sgRNA10-R | ACACCCCTGGACGTAACTTGACACCAATCC | 30 |

**Supplementary Table S2. The sequence of sgRNA**

| Name | Sequence (5’-3’) |
| --- | --- |
| IS6110-sgRNA1 | ACCGCUUCACUUAGAGUGAAGGUGGGCUGCUUGCAUCAGCCUAAUGUCGAGAAGUGCUUUCUUCGGAAAGUAACCCUCGAAACAAAGAAAGGAAUGCAAC**CGGUGGGGUGUCGAGUCGAU**UUUUAUUUU |
| IS6110-sgRNA2 | ACCGCUUCACUUAGAGUGAAGGUGGGCUGCUUGCAUCAGCCUAAUGUCGAGAAGUGCUUUCUUCGGAAAGUAACCCUCGAAACAAAGAAAGGAAUGCAAC**UCACCGUCGCCUACGCUCGC**UUUUAUUUU |

**（Continued Supplementary Table S2. The sequence of sgRNA）**

| IS6110-sgRNA3 | ACCGCUUCACUUAGAGUGAAGGUGGGCUGCUUGCAUCAGCCUAAUGUCGAGAAGUGCUUUCUUCGGAAAGUAACCCUCGAAACAAAGAAAGGAAUGCAAC**AUCAGCUCGGUCUUGUAUAG**UUUUAUUUU |
| --- | --- |
| IS6110-sgRNA4 | ACCGCUUCACUUAGAGUGAAGGUGGGCUGCUUGCAUCAGCCUAAUGUCGAGAAGUGCUUUCUUCGGAAAGUAACCCUCGAAACAAAGAAAGGAAUGCAAC**CGGUGCCCGCUUUGUGUGGC**UUUUAUUUU |
| IS6110-sgRNA5* | ACCGCUUCACUUAGAGUGAAGGUGGGCUGCUUGCAUCAGCCUAAUGUCGAGAAGUGCUUUCUUCGGAAAGUAACCCUCGAAACAAAGAAAGGAAUGCAAC**GUCAUCAGCCGUUCGACGGU**UUUUAUUUU |
| IS6110-sgRNA6* | ACCGCUUCACUUAGAGUGAAGGUGGGCUGCUUGCAUCAGCCUAAUGUCGAGAAGUGCUUUCUUCGGAAAGUAACCCUCGAAACAAAGAAAGGAAUGCAAC**GUCAACCCAGCACCUGCCAG**UUUUAUUUU |
| IS6110-sgRNA7 | ACCGCUUCACUUAGAGUGAAGGUGGGCUGCUUGCAUCAGCCUAAUGUCGAGAAGUGCUUUCUUCGGAAAGUAACCCUCGAAACAAAGAAAGGAAUGCAAC**GCCAUGAUCGACACUUGCGA**UUUUAUUUU |
| IS6110-sgRNA8 | ACCGCUUCACUUAGAGUGAAGGUGGGCUGCUUGCAUCAGCCUAAUGUCGAGAAGUGCUUUCUUCGGAAAGUAACCCUCGAAACAAAGAAAGGAAUGCAAC**UCGGUCAGAGCGUCGAGUAC**UUUUAUUUU |
| IS6110-sgRNA9 | ACCGCUUCACUUAGAGUGAAGGUGGGCUGCUUGCAUCAGCCUAAUGUCGAGAAGUGCUUUCUUCGGAAAGUAACCCUCGAAACAAAGAAAGGAAUGCAAC**GACCAGGCGCUCCAUCCGGC**UUUUAUUUU |
| IS6110-sgRNA10 | ACCGCUUCACUUAGAGUGAAGGUGGGCUGCUUGCAUCAGCCUAAUGUCGAGAAGUGCUUUCUUCGGAAAGUAACCCUCGAAACAAAGAAAGGAAUGCAAC**ACCACACCUUGGGGCACCUU**UUUUAUUUU |

**Supplementary Table S3. wild-type sgRNA (Cas12f1) and engineered sgRNA (Cas12f_ge4.1) sequences:**

| Name | Sequence (5’-3’) | Length(bp) |
| --- | --- | --- |
| Cas12f1 | CUUCACUGAUAAAGUGGAGAACCGCUUCACCAAAAGCUGUCCCUUAGGGGAUUAGAACUUGAGUGAAGGUGGGCUGCUUGCAUCAGCCUAAUGUCGAGAAGUGCUUUCUUCGGAAAGUAACCCUCGAAACAAAUUCAUUUUUCCUCUCCAAUUCUGCACAAgaaaGUUGCAGAACCCGAAUAGacgaaUGAAGGAAUGCAAC**GUCAUCAGCCGUUCGACGGU** | 222 |
| Cas12f_ge4.1 | ACCGCUUCACUUAGAGUGAAGGUGGGCUGCUUGCAUCAGCCUAAUGUCGAGAAGUGCUUUCUUCGGAAAGUAACCCUCGAAACAAAGAAAGGAAUGCAAC**GUCAUCAGCCGUUCGACGGU**UUUUAUUUU | 129 |

**Supplementary Table S4. ssDNA reporter**

| Name | Sequence (5’-3’) |
| --- | --- |
| FQ-6T | 5′-/6-FAM/TTTTTT/BHQ1/-3′ |
| FQ-9T | 5′-/6-FAM/TTTTTTTTT/BHQ1/-3′ |
| FQ-12T* | 5′-/6-FAM/TTTTTTTTTTTT/BHQ1/-3′ |
| FQ-18T | 5′-/6-FAM/TTTTTTTTTTTTTTTTTT/BHQ1/-3′ |
| FQ-21T  FB-12T* | 5′-/6-FAM/TTTTTTTTTTTTTTTTTTTTT/BHQ1/-3′  5′-/6-FAM/TTTTTTTTTTTT/Biotin/-3′ |
